# Quantifying the impact of heat on human physical work capacity; part IV: interactions between work duration and heat stress severity

**DOI:** 10.1007/s00484-022-02370-7

**Published:** 2022-10-05

**Authors:** James W. Smallcombe, Josh Foster, Simon G. Hodder, Ollie Jay, Andreas D. Flouris, George Havenith

**Affiliations:** 1 Environmental Ergonomics Research Centre, Loughborough University, Loughborough, LE11 3TU Leicestershire UK; 2 Thermal Ergonomics Laboratory, University of Sydney, Sydney, NSW Australia; 3 Institute for Exercise and Environmental Medicine, Texas Health Presbyterian Hospital and University of Texas Southwestern Medical Center, Dallas, TX USA; 4 FAME Laboratory, Department of Physical Education and Sport Science, University of Thessaly, Trikala, Greece

**Keywords:** Heatwave, Productivity, Workers, Climate, Hot

## Abstract

High workplace temperatures negatively impact physical work capacity (PWC). Although PWC loss models with heat based on 1-h exposures are available, it is unclear if further adjustments are required to accommodate repeated work/rest cycles over the course of a full work shift. Therefore, we examined the impact of heat stress exposure on human PWC during a simulated work shift consisting of six 1-h work-rest cycles. Nine healthy males completed six 50-min work bouts, separated by 10-min rest intervals and an extended lunch break, on four separate occasions: once in a cool environment (15 °C/50% RH) and in three different air temperature and relative humidity combinations (moderate, 35 °C/50% RH; hot, 40 °C/50% RH; and very hot, 40 °C/70%). To mimic moderate to heavy workload, work was performed on a treadmill at a fixed heart rate of 130 beats·min^−1^. During each work bout, PWC was quantified as the kilojoules expended above resting levels. Over the shift, work output per cycle decreased, even in the cool climate, with the biggest decrement after the lunch break and meal consumption. Expressing PWC relative to that achieved in the cool environment for the same work duration, there was an additional 5(± 4)%, 7(± 6)%, and 16(± 7)% decrease in PWC when work was performed across a full work shift for the moderate, hot, and very hot condition respectively, compared with 1-h projections. Empirical models to predict PWC based on the level of heat stress (Wet-Bulb Globe Temperature, Universal Thermal Climate Index, Psychrometric Wet-Bulb Temperature, Humidex, and Heat Index) and the number of work cycles performed are presented.

**Supplementary Information:**

The online version contains supplementary material available at 10.1007/s00484-022-02370-7.

## Introduction

A key consequence of environmental heat exposure is a reduction in human labour/physical work capacity (PWC) in industry sectors (Ioannou et al. 2017, 2022; Sahu et al. 2013; Wyndham, 1973). Recently, our group modelled the reduction in PWC using extensive empirical data collected across a wide range of environmental conditions with wide variations in temperature, humidity, solar radiation, air velocity, and aerobic fitness (Foster et al. 2021a, b, c and d). These studies were all based on measurements collected during 1-h work sessions in a wide variety of climates. The models are available for several common heat stress indices (e.g. Wet-Bulb Globe Temperature (WBGT), Universal Thermal Climate Index (UTCI), Wet-Bulb Temperature, Humidex, Heat Index) and can be used to estimate the impact of present and future environmental conditions on worker productivity in physical tasks, facilitating work planning during hot weather and allowing more accurate costing of climate change impact under different CO_2_ emission scenarios.

Our recent PWC models (work capacity in the heat expressed as the percentage of the total work possible in a cool climate) were developed from a large dataset that captured a wide range of heat stress severities. However, the 1-h work duration of the experiments may underestimate the physiological strain and impact associated with extended heat stress exposure (Kampmann and Bröde, 2022) and, thus, the work capacity loss in industry, where typical work shifts often last 6–8 h. For example, data derived from extended-duration occupational heat exposures indicate that workers often struggle to fully replace thermoregulatory sweat losses with adequate water consumption during the day, particularly when sweat rates are high (Kalkowsky and Kampmann, 2006). Given the known importance of hydration status in maintaining PWC in the heat (Cheung and McLellan, 1998; Marino et al. 2004), and an associated blunting of sweat secretion proportional to the level of intracellular dehydration (Cheuvront and Kenefick, 2014), progressive hypohydration throughout the day may exacerbate PWC losses beyond what our studies observed during shorter 1-h heat stress exposures. Additionally, with longer occupational heat stress exposures—even when rest breaks are permitted—heat may gradually accumulate in the body throughout the working day (Bröde et al. 2018, Kampmann and Bröde. 2022) contributing to elevated cardiovascular strain and further reduced work capacity.

Aside from extending our recently published PWC models, further research is also required to help better understand the thermoregulatory consequences of extended heat-stress exposure and to validate the conclusions drawn from short-duration experimental protocols. Only limited laboratory-based research describes thermo-physiological responses over prolonged heat-stress exposures (i.e. > 4 h; examples include but are not limited to Hellon et al. 1956; Lind and Hellon, 1957; Macartney et al. 2020; Wyndham et al. 1967). In contrast, often experimental paradigms rely on shorter experimental protocols during which individual or group responses to heat stress are examined (Foster et al. 2021b; Jay et al. 2011). Although the short-exposure experimental approach is pragmatic (full-day testing in a laboratory setting is extremely costly and time consuming), this may not fully capture the reality of more prolonged heat stress exposures, which are experienced commonly during occupational and recreational activities, and in heat wave scenarios.

The aim of the present study was to examine human thermoregulatory responses during an extended period of physical work in the heat and to quantify the loss of human physical work capacity across an extended work shift. An additional aim was to provide correction equations for our original 1-h work-based PWC model to enable physical work capacity to be modelled based on both heat stress severity and shift duration. We hypothesised that physical work capacity in the heat would be reduced as a function of the exposure duration and that this effect would increase with the heat stress severity.

## Method

### Ethical approval

Following approval from the Ethics Human Participants Sub-Committee at Loughborough University, nine healthy adult males volunteered for study participation. Participant characteristics are displayed in Table 1. Before admittance into the study, all participants completed a general health screen questionnaire to confirm suitability for study participation. All participants provided informed consent. Whilst a medical professional was not in attendance during experimental testing (in compliance with institutional requirements), all researchers were trained in emergency first aid and CPR. The criteria for terminating the testing (e.g. body core temperature limits) were set such that potential risk to health was low.

**Table 1:** Participant characteristics (*n* = 9 males)

	Mean (SD)	Range
Age (years)	23.7 (2.9)	19.7 to 27.7
Height (cm)	175.7 (4.5)	171.0 to 184.0
Body mass (kg)	70.8 (6.8)	60.9 to 79.3
BMI	23.0 (2.5)	20.7 to 27.0
Body fat (%)	13.7 (5.0)	8.8 to 26.3
Body surface area (m^2^)	1.86 (0.09)	1.71 to 1.93
$\overset.{\mathrm{V}}$O_2peak_ (mL·kg^−1^·min^−1^)	53.2 (7.4)	40.7 to 64.0
$\overset.{\mathrm{V}}$O_2peak_ (L·min^−1^)	3.7 (0.6)	2.7 to 4.7

#### Study design

A within-participants experimental design was employed with all participants visiting the laboratory on 5 separate occasions. Visit 1 consisted of an incremental submaximal exercise test used to estimate $\overset.V$O_2max_ and anthropometric assessment (height, mass, and body fat %).

Visits 2 to 5 were completed with a minimum of 7 days of separation. Participants completed a full work shift simulation trial on four separate occasions in the following environmental conditions (mean ± SD): 15.4 ± 0.7 °C, 47.7 ± 2.9% RH (cool, WBGT = 12.6 ± 0.5 °C) 34.6 ± 0.4 °C, 47.5 ± 1.5% RH (moderate, WBGT = 29.4 ± 0.4 °C); 39.7 ± 0.3 °C, 47.9 ± 1.7% RH (hot, WBGT = 33.4 ± 0.2 °C); and 39.6 ± 0.5 °C, 69.4 ± 3.9% RH (very hot, WBGT = 36.1 ± 0.2 °C). The mean air velocity across all trials was 0.32 m·s^−1^. Conditions were completed in a counterbalanced order. Using the same approach as described in detail in our previous work (Foster et al. 2021a, b, c), the 15 °C, 50% RH condition served as a cool (optimal) reference condition, to which performance in all subsequent hot trials was compared. The heat stress conditions for this study were chosen to elicit approximately 75%, 50%, and 25% of the total work achievable in the cool reference environment. Pilot tests indicated minimal additional loss in PWC (extended work shift vs 1 h) at WBGT < 29 °C.

#### Location and timeline

Data collection took place within the Environmental Ergonomics Research Centre at Loughborough University, in custom-made climatic chambers (TISS high-performance chambers, UK). Data was collected from January 2019 to September 2019. Whilst seasonal heat acclimatisation cannot be fully excluded, it has been shown to be minimal in prior work (Bain and Jay, 2011). In addition, the counterbalanced order of exposures should remove any impact of such a potential issue on the data. Furthermore, acclimation was minimised by allowing participants to complete a maximum of one experimental day per week.

#### Experimental controls

Experimental sessions for individual participants took place at the same time of day, with the goal to minimise any effects of circadian rhythms on results (Waterhouse et al. 2004). It is however worth noting that, apart from changes in absolute core temperature, physiological effector responses are unaffected by time of day (Ravanelli and Jay, 2020). Participants were asked to arrive hydrated before commencement of laboratory testing sessions (verified via urine specific gravity analysis), refrain from caffeine on the day of each trial (Hunt et al. 2021), and refrain from alcohol and vigorous exercise 24 h before each trial—compliance to these instructions was confirmed verbally upon arrival at the laboratory.

#### Preliminary trial (visit 1)

Anthropometric data were collected with participants dressed in T-shirt, shorts, and socks. Body mass (nude) was assessed using a digital scale (Metter Toledo kcc150, Metter Toledo, Leicester, UK; high-precision) and stature was determined to the nearest cm using a wall-mounted stadiometer (Holtain, Crosswell, UK). Body fat percentage was measured through bioelectrical impedance (TANITA Corporation, Tanita MC-780MA, Tokyo, Japan). Whilst not used in the subsequent data analysis, body fat percentage was determined to help characterise the morphology of our participant cohort.

An incremental submaximal exercise test (gradient-based) was performed in a room regulated at 18 °C, 40% RH using a motorised treadmill (Mercury Medical, h/p/cosmos sports & medical Gmbh, Germany). The test consisted of up to six 3-min stages. During the protocol, treadmill speed was fixed at 4.5 km·h^−1^ and its gradient was increased by 5% every 3 min until a steady-state heart rate of 85% of age-predicted maximum was attained (i.e. 220 − age). Expired gasses and heart rate were monitored continuously (Quark CPET, COSMED, Albano Laziale, Rome and Polar PE4000, Polar-electro, Kempele, Finland). Data on oxygen uptake and on heart rate in the submaximal treadmill test were extrapolated to maximum heart rate to obtain an estimate of maximal oxygen consumption (V̇O_2_ max) (ACSM, 2013).

#### Experimental protocol (visits 2–5)

**Fig. 1:**
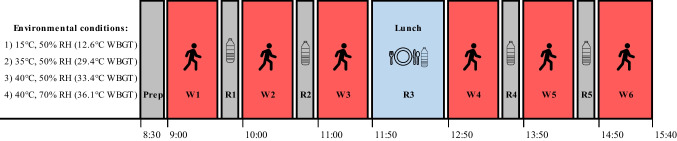
Schematic representation of the experimental protocol. After arrival and instrumentation (Prep), work cycle 1 (W1) began. All work cycles (W1–6) were 50 min in duration and were separated by a 10-min rest interval (R1–5) in a temperate environment (21 °C, 50% RH). During rest intervals, ad libitum water intake was permitted and quantified for later analysis. The standardised lunch period between work cycles 3 and 4 lasted 1 h. Each participant completed the protocol in all four environments listed in the figure, in a counterbalanced order

A schematic overview of the full-day experimental protocol is shown in Fig. 1. Upon arrival, participants inserted a rectal thermistor (VIAMED, Yorkshire, UK) to a depth of 10 cm past the anal sphincter to monitor internal (rectal) temperature, allowing continuous monitoring of core temperature. Then, they produced a urine sample for assessment of urine specific gravity. To ensure euhydration, participants were asked to drink ~ 500 mL of water if urine specific gravity was > 1.020 and, if so, to provide another urine sample after a further 20–30 min (Armstrong et al. 1994). To monitor skin temperature (*T*_skin_), skin thermistors (Grant Instruments Ltd, Corby, UK) were placed on the chest, arm (triceps), thigh, and calf. The mean *T*_skin_ was then calculated according to the Ramanathan equation (Ramanathan, 1964):

1 $${{\varvec{T}}}_{\mathbf{s}\mathbf{k}\mathbf{i}\mathbf{n}}=0.3{T}_{\mathrm{chest}}+0.3{T}_{\mathrm{arm}}+0.2{T}_{\mathrm{thigh}}+0.2{T}_{\mathrm{calf}}\left[^\circ \mathbf{C}\right]$$

Participants were lightly clothed, wearing underwear, standardised shorts, socks, and trainers. Using the reference tables in the International Standard (ISO9920, 2009), the clothing ensemble corresponded to an intrinsic thermal insulation of 0.04 m^2^·K·W^−1^ (0.26 Clo). Using Eq. 31 in the International Standard, the evaporative resistance was estimated to be 0.007 m^2^·kPa·W^−1^.

Each hour of the work cycle consisted of 50 min of treadmill walking at either the cool, moderate, hot, or very hot condition, interspersed with 10 min of rest. The selection of this work-to-rest ratio was guided by the intermittent work profile proposed by Bröde and colleagues (2018) and further informed by extensive pilot testing, during which it became evident that 10-min rest intervals to be the minimum duration of rest required to allow the completion of six 50-min work bouts at a heart rate of 130 beats·min^−1^ without unacceptable levels of fatigue. Additionally, these rest breaks allowed for ad libitum water consumption, as is a common requirement in many industries. The treadmill was programmed to automatically change its speed (maximum 6 km·h^−1^) and grade to ensure a constant heart rate of 130 beats·min^−1^ was maintained throughout each work cycle. The full rationale for the selection of the 130 beats·min^−1^ heart rate work intensity—which represents the demarcation between moderate and heavy strain (Andersen 1978) and is considered to be the maximal acceptable workload for sustained work periods (Bernard and Kenney 1994)—is provided in our companion paper (Foster et al. 2021b), to which interested readers are directed. The speed and grade data were used to estimate the energy generated (above resting) in each 50-min work cycle using the following equation (Ludlow and Weyland, 2017):

2 $$\mathrm{Work}\;\mathrm{EE}=\sum\nolimits_{\mathrm t=1}^{50}\left[0.32\cdot\mathrm G(\mathrm t)+3.28+\left(1+0.19\cdot\mathrm G(\mathrm t)\right)\cdot\left(2.66\cdot{\mathrm v(\mathrm t)}^2\right)\right]\cdot\left[19.61+\frac{\mathrm{RQ}(\mathrm t)-0.707}{0.293}\cdot1.51\right]\lbrack\mathrm{kJ}\rbrack$$

where *G*(*t*) is the slope of the treadmill expressed in percent grade at time *t*, *v*(*t*) is velocity of walking expressed in metres per second at the same time point, RQ(*t*) is respiratory quotient which was assumed to be 0.85 (Cramer and Jay, 2019). The summation function (∑) denotes that the output of the equation is summed every 1 min (*t* = 1) until the 50-min work bout was completed (in some cases where HR increased above 130 beats·min ^−1^ when resting, the work bout was ended early and the final integrated work output used), accounting for the change in each variable over time. Part 1 (first square bracket) of the equation (Ludlow and Weyand, 2017) calculates the net volume of oxygen consumed ($\overset.{\mathrm V}$O_2-net_, in mL·kg body mass^−1^·min^−1^) to fuel exercise, i.e. not including resting $\overset.{\mathrm V}$O_2_. Part 2 of the equation (second square bracket) converts the former into kJ min^−1^. The cumulative EE over all six 50-min work bouts was used to calculate the total EE (in kJ) above resting over the whole shift.

Physical work capacity in the heat was calculated by:

3 $$\mathrm{Physical}\;\mathrm{work}\;\mathrm{capacity}\;(\mathrm{\it i}\; \mathrm{cycles})=\left(\frac{\int_1^iHot_{\mathrm{kJ}}}{\int_1^iCool_{\mathrm{kJ}}}\right)\times100\left(\%\right)$$

where *cycles* = the number of 1-h work cycles, *Cool*_kJ_ is the total energy generated (kilojoules, kJ) above resting metabolism in the cool reference condition, and *Hot*_kJ_ is the total energy generated above resting metabolism in each heat stress trial. The cumulative value for *Hot*_kJ_ and *Cool*_kJ_ was used to calculate how PWC is affected by work duration. For example, 1-h PWC is calculated by using the value of *Hot*_kJ_ and *Cool*_kJ_ after one work cycle, whereas 6-h PWC is calculated by using the cumulative value of both *Hot*_kJ_ and *Cool*_kJ_ after 6 work cycles (i.e. full work shift).

Upon completion of each work cycle, participants exited the environmental chamber, nude body mass was quantified to determine whole-body sweat loss, and participants undertook 10 min of seated rest in temperate conditions (21 °C, 50% RH). During this rest period, ad libitum water intake was permitted, and the volume of water consumed and urinated was recorded. After 10 min of rest, participants returned to environmental chamber for the completion of the next scheduled work cycle. This process was repeated until a total of 6 work cycles were completed. Six work cycles mimicked a 7-h workday (including a 1-h lunch break).

#### Lunch break

Between the third and fourth work cycle participants were given a 1-h lunch break, during which they rested in temperate conditions (21 °C, 50% RH) and consumed a standardised lunch meal consisting of cheese and tomato pasta, cereal bar, banana, and an isotonic carbohydrate sports drink. In total, the lunch provided 925 kcal; 156 g of carbohydrate; 27 g of protein; 22 g of fat. Ad libitum water intake was also permitted, and quantified, during the lunchbreak. We consider the standardisation of food to be an important feature of our study design. During piloting, we observed that the ingestion (and digestion) of food exerted a profound impact on heart rate responses during subsequent work performed in the heat. Given the fixed heart rate protocol employed in the current study, it was important that any additional cardiovascular strain imposed by the consumption of lunch was consistent across experimental trials and between participants. Failure to standardise the lunchtime meals would have increased both the within- and between-participant variation in performance in the afternoon work bouts to an unacceptable level (especially considering our modest sample size). The standardised meal was designed to be palatable to all participants (i.e. did not contain meat, nuts/other allergens etc.) and was representative of a ‘typical’ meal (i.e. balanced in macronutrient content), whilst also providing sufficient energy (925 kcal) to enable physical work of extended duration.

The volume of isotonic sports drink (Lucozade Ribena Suntory Limited) was considered when calculating fluid balance (Fig. 5), whilst the water content of the food (322.4 g) was also accounted for (estimates derived from the CompEat Pro 5.8.0 computerised food tables; Nutrition Systems, London, UK).

#### Calculation of the heat stress indices

##### Wet-bulb globe temperature

The WBGT (outdoor equation) was determined using a WBGT monitor (QUESTemp model 34, TSI Incorporated), which measured ambient temperature *T*_*a*_, globe temperature *T*_*g*_, And Natural Wet-Bulb Temperature *T*_*nwb*_*.* The value used for modelling was the average over the course of each work cycle. As in the climatic chamber *T*_*a*_ equals *T*_*g*_, the outcome of the outdoor and indoor WBGT equation deliver the same result.

#### Aspirated (psychrometric) wet-bulb temperature

Aspirated wet-bulb temperature was calculated based on the formula provided by Stull (2011):

4 $$\begin{array}{l}T_{wb}=T_a\times\mathrm{atan}\left[0.151977\left(Rh+8.313659\right)^\frac12\right]+\mathrm{atan}\left(T_a+RH\right)-\\\mathrm{atan}\;\left(RH-1.676331\right)+0.00391838\left(RH\right)^{3/2}\times\mathrm{atan}\;\left(0.023101RH\right)-4.686035\lbrack^\circ\mathrm C\rbrack\end{array}$$

where *T*_*a*_ is air temperature in degrees Celsius and RH is relative humidity in % (0–100).

#### Universal Thermal Climate Index

An excel calculator was used to determine the UTCI (www.climatechip.org/excel-wbgt-calculator), using the published regression polynomial of UTCI (Bröde et al. 2012). The input values used for the calculation were *T*_a_, RH, mean radiant temperature and air velocity (converted to 10-m-high value as required by UTCI).

#### Humidex

The Humidex calculation was based on (Masterton and Richardson, 1979; Rana et al. 2013):

5 $$Humidex={T}_{\mathrm{a}}+\frac{5}{9}\left(\left[6.112\times {10}^{\left(\frac{7.5{T}_{\mathrm{a}}}{237.7+{T}_{\mathrm{a}}}\right)}\times \frac{RH}{100}\right]-10\right) [^\circ \mathrm{C}]$$

#### Heat Index

The Heat Index was calculated based on (Rothfusz, 1990):

6 $$\begin{array}{l}\mathrm{Heat}\;\mathrm{Index}=-42.379+2.04901523T_{\mathrm a}+10.14333127RH-\\0.22475541T_{\mathrm a}\cdot RH-6.83783\times10^{-3}T_{\mathrm a}^2-5.481717\times10^{-2}RH^2+\\1.22874\times10^{-3}T_{\mathrm a}^2\cdot RH+8.5282\times10^{-4}T_{\mathrm a}\cdot RH^2-1.99\times10^{-6}T_{\mathrm a}^2\cdot Rh^2\lbrack^\circ\mathrm F\rbrack\end{array}$$

where *T*_a_ is in degrees Fahrenheit and RH is in % (0–100). The Heat Index in Fahrenheit (*HI*_F_) was converted to degrees Celsius by:

7 $$Heat\;Index=\left(HI_{\mathrm F}-32\right)\times\frac59\lbrack^\circ\mathrm C\rbrack$$

#### Statistical analyses

Statistical analyses were performed using IBM SPSS version 27. Differences in absolute kJ energy output, thermometric variables (average core, skin, and body temperature for each cycle), and perceptual variables (average rating of perceived exertion, thermal sensation, thermal comfort for each cycle) were analysed using a two-way repeated measures ANOVA. The independent variables were heat stress condition (4 levels; cool, moderate, hot, very hot) and work cycle (6 levels; W1, W2, W3, W4, W5 and W6). The impact of whole-body sweat loss on net fluid loss over the day was analysed using a mixed model with whole-body sweat loss (WBSL) as fixed, and participant as random factor. For the random factor, both the slope and intercept were variable. The independent effect of the lunch break and meal on the reduction in PWC (absolute kilojoules) was also examined, with the same 4 levels of heat stress conditions, but with two levels of work cycle (W3 vs W4). The influence of heat stress on the relative change in PWC (% reduction from W3 to W4) caused by the lunch break and meal consumption was tested using a one-way ANOVA. The two-tailed alpha value of significance testing was set as *p* < 0.05.

Correction equations for the impact of work duration on PWC were developed using linear regression of additional PWC loss due to longer work versus the work time, expressed in the number of work cycles. The additive correction is, by definition, zero for a single work cycle, as the original model (Foster et al. 2021b) is based on that. The model describes the additional impact over a range of two to six work cycles. Corrections are developed for each of the hot climates, and calculations are presented showing interpolations between the different levels of heat stress.

## Results

### Performance responses

#### Physical work capacity

**Fig. 2:**
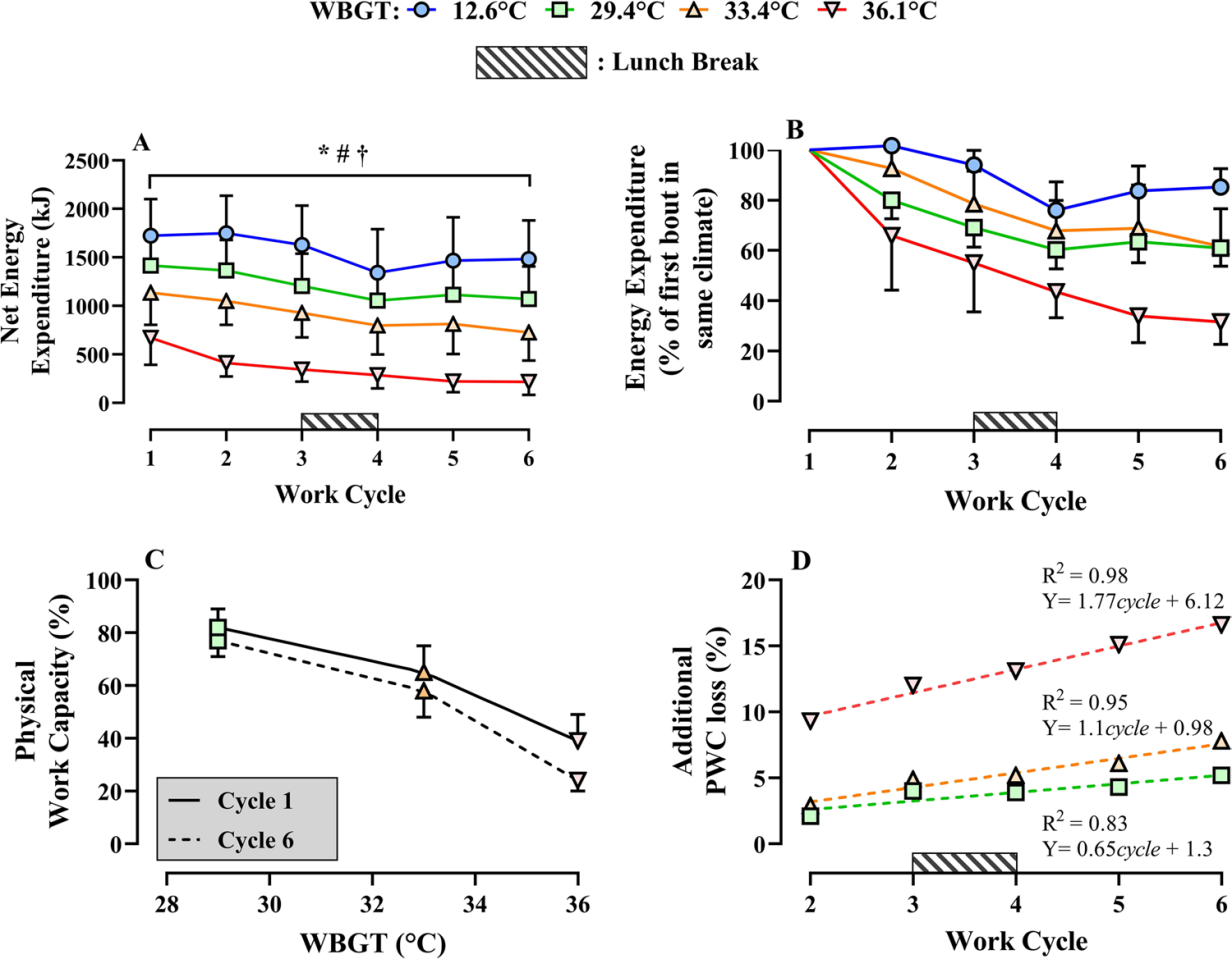
Panel **A** shows the absolute energy expenditure above resting, achieved in each work cycle separated by Wet-Bulb Globe Temperature (WBGT). The asterisk denotes the main effect for WBGT, the number sign denotes the main effect for work cycle, and the dagger denotes the interaction effect (all *p* < 0.01). Panel **B** shows, as a percentage, the energy expenditure achieved in each individual work cycle (W2–6) compared to the first work cycle (W1) in the same climate condition. Panel **C** shows the physical work capacity (PWC%) achieved relative to the PWC in the cool climate (12.6 °C WBGT) for the same work duration, assessed after 1 (solid line) or the total for 6 (dashed line) work cycles. The difference shows the extra loss over a full work shift versus a single cycle of work. Panel **D** shows these same differences seen in panel **C**, for the cumulative effect of 2 to 6 work cycles. The data in panel **D** were subsequently used to form correction equations, for the prediction of PWC based on the interaction between heat stress severity and work cycle duration (number of work cycles). Raw data are available in supplementary Table 1

For the cool condition, work output remained quite stable in the morning, but decreased after lunch and then showed a partial recovery in cycles 5 and 6 (Fig. 2 and supplementary Table 1). For the full work shift trial, increasing heat stress severity (expressed as WBGT) decreased whole work-shift PWC (main effect for WBGT, F_3,24_ = 86.12, *p* < 0.001, *η*_p_^2^ = 0.92). PWC also decreased with increasing number of work cycles (main effect for work cycle, F_5,40_ = 57.69, *p* < 0.001, *η*_p_^2^ = 0.88). There was an interaction between WBGT and work cycle number (interaction effect, F_15,120_ = 5.68, *p* < 0.001, *η*_p_^2^ = 0.42) with the impact of work duration on PWC becoming larger with increasing WBGT.

### Food intake and physical work output

**Fig. 3:**
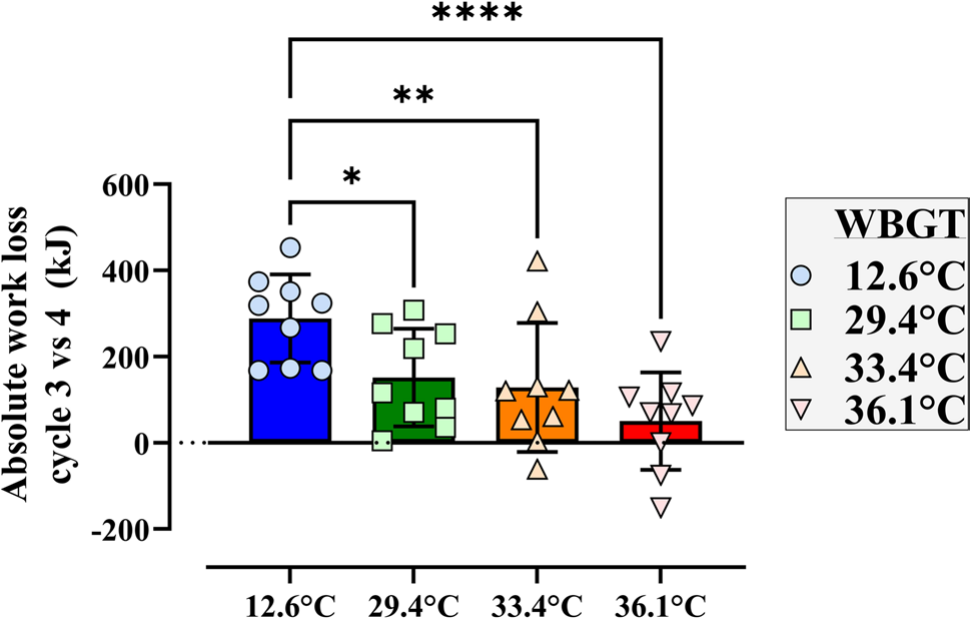
Effect of the lunch break and meal consumption on the absolute loss in work output, shown through the difference between cycle 3 and 4. *p < 0.05; **p < 0.01; ****p < 0.0001; ns, not significant. The error bars indicate the SD of the data. All values except the 36.1 °C were significantly different from zero

Despite the longer rest time during the lunch break, work output in the first session after lunch (W4) was lower compared to the work cycle immediately before lunch (W3, F_1,8_ = 28.30, *p* < 0.01, *η*_p_^2^ = 0.78, Fig. 3). As heat stress severity increased, the additional lunch-induced reduction in PWC lessened, with a strong interaction effect observed (F_3,24_ = 9.43, *p* < 0.001, *η*_p_^2^ = 0.54) with the difference pre- to post-lunch becoming non-significant at 36.5 °C WBGT (*p* > 0.05). A recovery of work capacity was observed during the second work cycle after lunch, but this recovery was diminished with increasing heat stress severity and was absent at the highest heat stress level (Fig. 3).

### Thermoregulatory responses

**Fig. 4:**
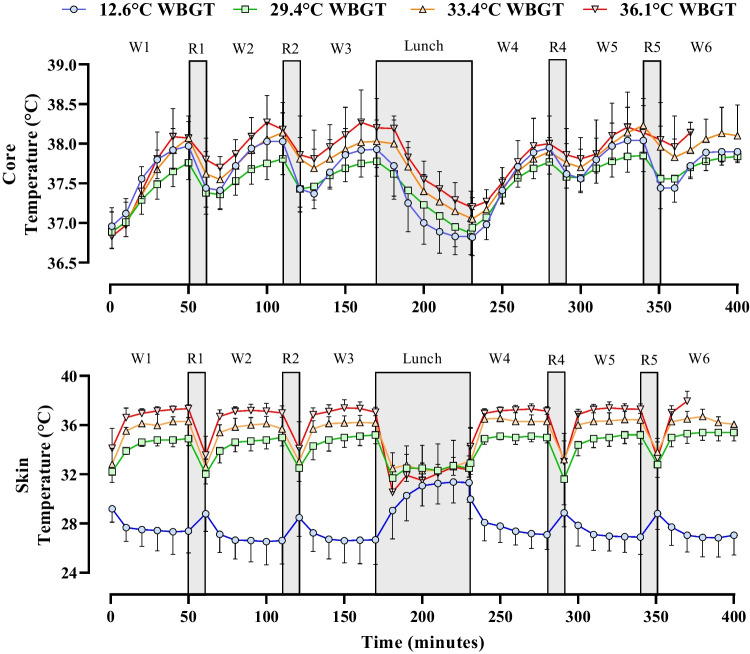
Rectal and skin temperature response throughout the full work shift protocol, separated by Wet-Bulb Globe Temperature (WBGT) condition. W, work cycle; R, rest. N.B limited data are presented for W6 at 36.1 °C WBGT as when participants became unable to perform any work during the final work cycle (i.e. no work was possible without exceeding 130 beats·min^−1^) they exited the chamber and the experimental session was terminated. Error bars indicate the SD of the data, which represent the average HR values of all participants recorded at 10-min intervals over the course of the day. The drop in core temperature before the rest period starts (e.g. W2 at 36.1 °C WBGT) is due to some participants stopping work as their HR exceeded 130 beats·min ^−1^ even at rest

Rectal and skin temperature responses across the work shift are shown in Fig. 4 and in supplementary Table S2. Comparisons between work cycle 1 and work cycle 6 for each condition are shown in supplementary Figure S1.

### Rectal (core) temperature

Whilst not for all individual heat increments, across the board rectal temperature increased with increasing heat stress severity (main effect for WBGT, F_3,24_ = 8.25, *p* < 0.01, *η*_p_^2^ = 0.51) and with increasing work duration (main effect for work cycle, F_5,40_ = 18.88, *p* < 0.001, *η*_p_^2^ = 0.70). The impact of work duration (work cycle number) increased with heat stress severity with a significant interaction effect observed (F_15,120_ = 2.92, *p* < 0.01, *η*_p_^2^ = 0.27).

### Skin temperature

Skin temperature increased with heat stress severity (main effect for WBGT, F_3,24_ = 266.92, *p* < 0.001, *η*_p_^2^ = 0.97). Whilst there was a main effect for work cycle (F_5,40_ = 3.11, *p* < 0.05, *η*_p_^2^ = 0.28), post hoc tests show no differences in *T*_skin_ over time, for any heat stress condition. There was a significant interaction effect observed (F_15,120_ = 4.49, *p* < 0.001, *η*_p_^2^ = 0.36), suggesting that *T*_skin_ changed with WBGT and work cycle. At each cycle number, *T*_skin_ was different across all heat conditions, except for no difference between WBGT 34.1 and 36.5 °C in the final work cycle (W6) (*p* > 0.05). Average *T*_skin_ values for each work cycle and WBGT condition are shown in supplementary Table S2.

### Whole-body sweat loss and fluid intake

Mean whole-body sweat losses and fluid intake (drinking + meal) across work cycles are presented in supplementary Table S3.

**Fig. 5:**
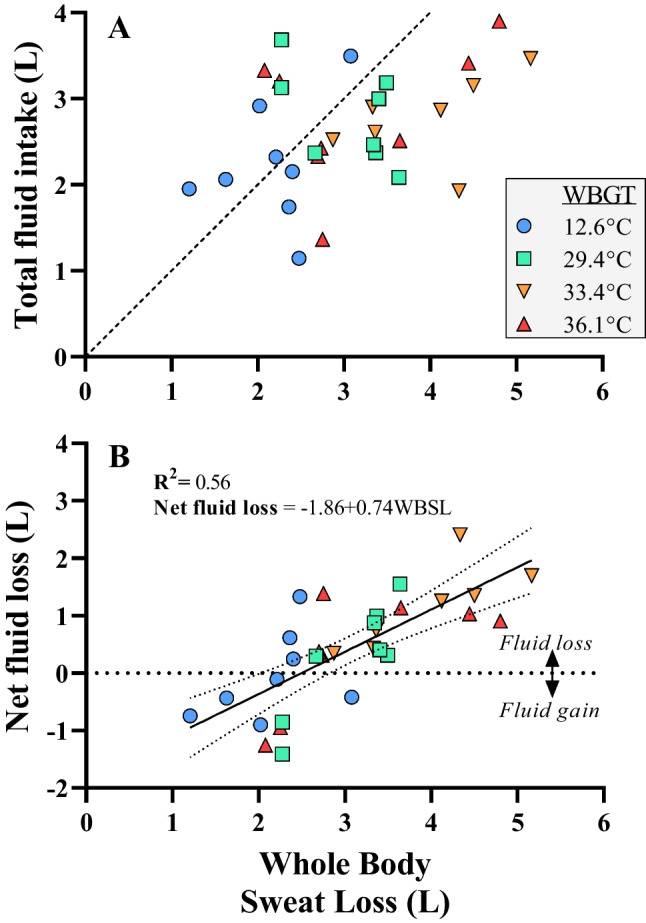
**A** The relationship between total fluid intake and whole-body sweat loss and **B** net fluid loss and whole-body sweat loss over the course of an extended work shift. Net fluid loss increases linearly with whole-body sweat loss, despite ad libitum water intake. Regression analysis using WBSL as fixed and participant as random factor

The analysis of net fluid loss across the day corrected for whole-body sweat loss across that period (and corrected for individually different responses) resulted in the relationship depicted in Fig. 5 (Net fluid loss =  − 1.86 + 0.74WBSL). The explained variance based on the fixed factor WBSL was calculated as 56%.

Whole-body sweat loss (WBSL) increased with heat stress severity (WBGT) (F_3,24_ = 6.26, *p* < 0.05, *η*_p_^2^ = 0.44) and work duration (number of work cycles) (F_5,40_ = 9.60, *p* < 0.01, *η*_p_^2^ = 0.55), but there was no interaction effect between WBGT and work duration (F_4,27_ = 2.108, p > 0.05, *η*_p_^2^ = 0.21).

We found a strong relationship between total net fluid loss (i.e. fluid deficit = WBSL-water uptake) and gross WBSL over the work shift (*R*^2^ = 0.56). When WBSL was ≤ 2 L across the working day, that water loss was sufficiently replaced by water intake, causing no net fluid loss or even a mass gain. However, when WBSL was ≥ 2 L across the day, a deficit developed that increased in a linear fashion at ~ 0.74 L for every 1 L of sweat secreted above 2 L. The relationship between net fluid deficit and WBSL is shown in Fig. 5.

### Modelling physical work capacity

The reduction in PWC observed consists of two components: (1) the reduction due to the heat as described in the previously published 1-h data (Foster et al. 2021b) and (2) the additional reduction observed when work duration is extended beyond a single work bout. The latter is represented in Fig. 2D, showing the extra reduction due to heat when the number of work cycles is extended. The regression equations in Fig. 2D are used to define corrections for the interaction of extended work time and environmental heat load. It was arbitrarily chosen to use a linear interpolation for climate values between the regression lines at the different heat load levels. For the reference condition and temperatures below, this is defined as zero, whilst for heat stress levels above the highest tested the values are limited to the highest values obtained, as no data are available above this. The corrections are presented in Table 2, based on either WBGT, UTCI, *T*_wb_, Humidex, and Heat Index. Column two shows the basic correction based on the 1-h data (Foster et al. 2021b) and column three the additional correction needed when two or more work cycles are performed.

**Table 2:** Correction equations for calculation of physical work capacity (PWC) based on work duration for different heat stress indices. These corrections should only be applied if two or more work cycles are completed. The values generated from PWC_corr_ (3rd column) should be subtracted from the 1-h projections in our companion paper (2nd column; Foster et al. 2021b)

Heat stress metric and range (°C)	PWCref: original physical work capacity equation for one 1-h work cycle (Foster et al. 2021b)	PWCcorr: correction value for additional loss in physical work capacity depending on the number of 1-h work cycles, which needs to be subtracted from PWCref in column two. This correction only applies for 2 to 6 work cycles	
Wet-Bulb Globe Temperature (WBGT)			
IF WBGT < 12.6	$PWCref=\frac{100}{1+\left(\frac{33.63}{WBGT}\right)^{-6.33}}$	$PWCcorr=$ 0	Equation (8)
IF 12.6 < WBGT ≤ 29.4		$PWCcorr=\left(\frac{WBGT-12.6}{16.8}\times [0.65cycles+1.3]\right)$	Equation (9)
IF 29.4 < WBGT ≤ 33.4		$PWCcorr=\left(\frac{33.4-WBGT}{4.0}\times [0.65cycles+1.3]\right)+ \left(\frac{WBGT-29.4}{4.0}\times [1.1cycles+0.98]\right)$	Equation (10)
IF 33.4 < WBGT ≤ 36.1		$PWCcorr=\left(\frac{36.1-WBGT}{2.7}\times [1.1cycles+0.98]\right)+ \left(\frac{WBGT-33.4}{2.7}\times [1.77cycles+6.12]\right)$	Equation (11)
IF WBGT > 36.1		$PWCcorr= \left(1.77cycles+6.12\right)$	Equation (12)
Universal Thermal Climate Index (UTCI)			
IF UTCI < 15.8	$PWCref= \frac{100}{1+{\left(\frac{45.33}{UTCI}\right)}^{-4.30}}$	$PWCcorr=$ 0	Equation (13)
IF 15.8 < UTCI ≤ 35.6		$PWCcorr=\left(\frac{UTCI-15.8}{19.8}\times [0.65cycles+1.3]\right)$	Equation (14)
IF 35.6 < UTCI ≤ 42.5		$PWCcorr=\left(\frac{42.5-UTCI}{6.9}\times [0.65cycles+1.3]\right)+ \left(\frac{UTCI-35.6}{6.9}\times [1.1cycles+0.98]\right)$	Equation (15)
IF 42.5 < UTCI ≤ 50.8		$PWCcorr=\left(\frac{50.8-UTCI}{8.3}\times [1.1cycles+0.98]\right)+ \left(\frac{UTCI-42.5}{8.3}\times [1.77cycles+6.12]\right)$	Equation (16)
IF UTCI > 50.8		$PWCcorr= \left(1.77cycles+6.12\right)$	Equation (17)
Aspirated/Psychrometric Wet-Bulb Temperature (*T*_wb_)			
IF *T*_wb_ < 11.3	$PWCref= \frac{100}{1+{\left(\frac{30.98}{{T}_{wb}}\right)}^{-5.90}}$	$PWCcorr=$ 0	
IF 11.3 < *T*_wb_ ≤ 27.1		$PWCcorr=\left(\frac{{T}_{wb}-11.3}{15.8}\times [0.65cycles+1.3]\right)$	Equation (18)
IF 27.1 < *T*_wb_ ≤ 30.8		$PWCcorr=\left(\frac{30.8-{T}_{wb}}{3.7}\times [0.65cycles+1.3]\right)+ \left(\frac{{T}_{wb}-27.1}{3.7}\times [1.1cycles+0.98]\right)$	Equation (19)
IF 30.8 < *T*_wb_ ≤ 34.6		$PWCcorr=\left(\frac{34.6-{T}_{wb}}{3.8}\times [1.1cycles+0.98]\right)+ \left(\frac{{T}_{wb}-30.8}{3.8}\times [1.77cycles+6.12]\right)$	Equation (20)
IF *T*_wb_ > 34.6		$PWCcorr= \left(1.77cycles+6.12\right)$	Equation (21)
Humidex			
IF Humidex < 15.4	${{P}}{{W}}{{C}}{{r}}{{e}}{{f}}= \frac{100}{1+{\left(\frac{54.50}{Humidex}\right)}^{-4.10}}$	$PWCcorr=$ 0	Equation (22)
IF 15.4 < Humidex ≤ 43		$PWCcorr=\left(\frac{Humidex-15.4}{27.6}\times [0.65cycles+1.3]\right)$	Equation (23)
IF 43 < Humidex ≤ 53		$PWCcorr=\left(\frac{53-Humidex}{10}\times [0.65cycles+1.3]\right)+ \left(\frac{Humidex-43}{10}\times [1.1cycles+0.98]\right)$	Equation (24)
IF 53 < Humidex ≤ 62		$PWCcorr=\left(\frac{62-Humidex}{9}\times [1.1cycles+0.98]\right)+ \left(\frac{Humidex-53}{9}\times [1.77cycles+6.12]\right)$	Equation (25)
IF Humidex > 62		$PWCcorr= \left(1.77cycles+6.12\right)$	
Heat Index			
IF Heat Index < 15	$PWCref= \frac{100}{1+{\left(\frac{55.47}{HeatIndex}\right)}^{-2.90}}$	$PWCcorr=$ 0	Equation (26)
IF 15 < Heat Index ≤ 39.8		$PWCcorr=\left(\frac{HeatIndex-15}{24.8}\times [0.65cycles+1.3]\right)$	Equation (27)
IF 39.8 < Heat Index ≤ 52.0		$PWCcorr=\left(\frac{52-HeatIndex}{12.2}\times [0.65cycles+1.3]\right)+ \left(\frac{HeatIndex-39.8}{12.2}\times [1.1cycles+0.98]\right)$	Equation (28)
IF 52.0 < Heat Index ≤ 67.7		$PWCcorr=\left(\frac{67.7-HeatIndex}{15.7}\times [1.1cycles+0.98]\right)+ \left(\frac{HeatIndex-52}{15.7}\times [1.77cycles+6.12]\right)$	Equation (29)
IF Heat Index > 67.7		$PWCcorr= \left(1.77cycles+6.12\right)$	Equation (30)

**Fig. 6:**
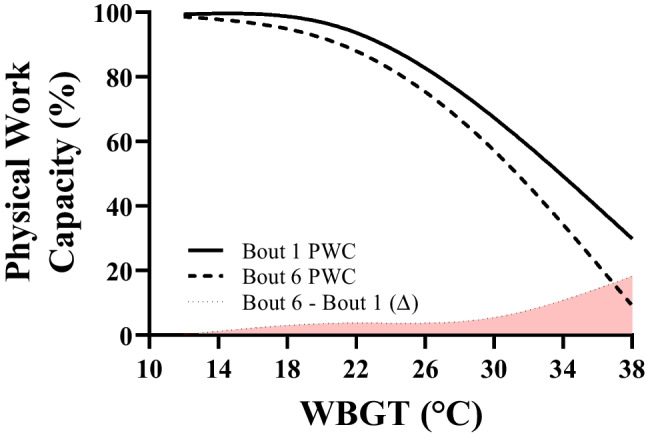
The interaction between Physical Work Capacity (PWC), Wet-Bulb Globe Temperature (WBGT), and work duration (cycle number). The solid line represents PWC based on our 1-h projections published recently (Foster et al, 2021a, b, c, d). The dashed line shows PWC at cycle 6, based on the current study and the correction equations in Table 2. The red area fill shows the size of the difference between the two models

Using these correction equations, PWC can now be predicted for different work durations from:

31 $${Physical\;Work\;Capacity}_{cycles}=PW{C}_{ref}- PW{C}_{corr, cycles}$$

where ‘cycles’ stands for the number of work cycles performed, *PWC*_*ref*_ (column two in Table 2) should be taken from the models presented in our companion paper (Foster et al. 2021b), and *PWC*_*corr*_, only applicable if more than one cycle is performed, is shown in Table 2, column three. An example output using WBGT as the heat stress assessment metric is shown below in Fig. 6.

### Example calculation

Below we provide an example calculation based on the format set out in Table 2 of this paper. The example is based on WBGT, in which the correction factor (*PWC*_*corr*_) is shown in Table 2. The calculation below assumed a WBGT of 32 °C and six work cycles being performed.

32 $$Physical\;work\;capacity\;(\boldsymbol{\mathit\%})=\left[\frac{100}{1+\left(\frac{33.63}{32}\right)^{-6.33}}\right]-\left[\left(\frac{33.4-32}{4.0}\times\lbrack\left(0.65\times6\right)+1.3\rbrack\right)+\left(\frac{32-29.4}{4.0}\times\lbrack\left(1.1\times6\right)+0.98\rbrack\right)\right]$$

Using the original model based on 1 h of work (i.e. without the correction), PWC would be 58%, which is reduced by a further 6.7% when an extended shift is worked. As such, using the above example, PWC is calculated as 51%. Note the original model (*PWC*_*ref*_, the left square bracket) should be taken from column 2 of Table 2.

## Discussion

The current study examined how work duration and heat stress severity interact with human physical work capacity (PWC) in work paced by constant cardiovascular strain. Considering the data obtained in the cool reference climate (12.6 °C WBGT), PWC in the sixth hourly work cycle is reduced by 17% compared to the work done in the first hour. The largest negative impact on PWC in the cool climate was observed in the work bout immediately following the lunch break, which may plausibly be explained by the additional blood flow competition imposed by food ingestion and digestion. Having studied how this baseline reduction in relative PWC changes when heat stress is added, we now provide empirical evidence that, apart from the additional heat-induced impairment of PWC increasing nonlinearly with heat stress severity as observed previously (Foster et al. 2021a, c, d) it is also dependent on the exposure duration/work time. Using WBGT as an example index, we found only modest additional effects of exposure time (< 5%) on cumulative PWC at WBGT ≤ 30 °C, but the impact grew progressively when WBGT > 30 °C. Thus, for most working conditions—including those that fall below the International Standard (ISO7243 2017) reference limit WBGT values for physical work—the obtained PWC losses in 1-h experiments (Foster et al. 2021a, b, c, d) provide an acceptable approximation (i.e. within 5%) for whole working days, but for WBGT > 30 °C, the additional corrections provided in this paper should be used. Correction equations are generated such that PWC can be predicted based on (a) one of five different heat stress metrics (WBGT, UTCI, *T*_wb_, Heat Index, or Humidex) and (b) exposure time.

The progressive reductions in PWC observed across the work shift appear primarily driven by the lunch break and associated meal consumption (see Fig. 3). For example, after the lunch break, PWC dropped by ~ 20% of the pre-lunch cycle value in all conditions (i.e. cycle 3 vs cycle 4, where lunch was consumed in between these cycles). Whilst strictly spoken in the present experimental design we cannot differentiate between the effect of the break and the effect of the food, the observed drop in PWC may be plausibly explained by the well-established phenomenon termed postprandial hyperaemia which is characterised by increased gastrointestinal blood flow observed after meal consumption (Madsen et al. 2006). In the context of the current experimental design (i.e. clamped cardiovascular strain/heart rate model), redistribution of blood towards the digestive system provides additional competition for a limited cardiac output, necessitating a reduction of blood flow to active musculature resulting in a reduction of PWC. Figure 2 demonstrates that PWC was maintained quite well in the cool climate *prior* to the lunch break, suggesting little influence of ‘cumulative’ strain on PWC. In the warmer conditions, a progressive reduction in PWC was noted *prior* to food ingestion, suggesting a more direct effect of prolonged heat exposure on PWC (compared with 1-h cycles). In the current study, consistent and progressive increases in rectal (but not skin) temperature were observed across the six work cycles, in all conditions apart from the cool reference (see Fig. 4 and supplementary Fig. 1). Furthermore, water deficits (water loss through sweat not countered by the ad libitum fluid intake in the breaks) were noted *only* in the warm conditions, as shown in Fig. 5. Throughout repeated work cycles, the combination of cumulative thermal strain and dehydration likely mediated the reductions in PWC prior to the lunch break. The exponential loss in PWC in the hottest condition (WBGT = 36.1 °C) is potentially further amplified by a reduction in stroke volume mediated by excessive venous pooling (i.e. reducing left ventricular end-diastolic volume). The progressive increases in core temperature observed with repeated cycles of work have important implications for occupational heat safety management, as a propensity for cycle-by-cycle heat storage may further predispose workers to hyperthermia, and potentially heat-related morbidity, during the latter part of a work shift (Flouris et al. 2018). Whilst it is acknowledged that the heat stress conditions used in the current study exceed the International Standard (ISO7243) reference WGBT values for physical work, it should be noted that workers in many global regions are already faced with very severe environmental conditions in which they are either expected—or necessitated—to work. Furthermore, current climate change projections indicate that the severity of future extreme heat events will continue to worsen. In such future climate scenarios, it is very likely that the current ISO limit values will be regularly exceeded.

Compared with short exposures (1–2 h), relatively few studies have investigated the impact of prolonged heat exposure on work performance and physiological strain across multiple hours. Hellon et al. (1956) reported a gradual rise in rectal temperature with successive simulated work bouts (stepping at a fixed rate) in both younger and older men over the course of 4 h. Although rectal temperature was similar in both age groups, the older group were working at a greater absolute heart rate by ~ 10 beats·min^−1^ throughout the protocol. Similar physiological responses were also reported during passive heat stress exposure across a range of effective temperatures with progressive increases in core temperature and cardiovascular strain observed across 180-min (Lind and Hellon, 1957). A progressive rise in core temperature was also observed during the first 3 h of fixed-rate work at 32.2 °C effective temperature, although rectal temperature plateaued between the third and fifth hours (Wyndham et al. 1960). During fixed intensity treadmill work at 32.5 °C WBGT, rectal temperature and heart rate did not progressively increase with exposure duration (Anderson et al. 2022). Whilst important differences in exercise prescription (i.e. fixed vs self-paced work simulation) preclude direct study comparison, these findings broadly align with those of the current study as we observed only modest increases in rectal temperature across the protocol. The modest increase in rectal temperature can be explained by the downregulation of work output in the constant cardiovascular strain model used in the present study, representing pacing behaviour. Importantly, in young adults, previous research reported no impairment of heat loss and no additional heat storage on the day after performing 7.5 h of fixed-paced physical work in hot dry conditions (Notley et al. 2018b). However, impairments in heat loss on day two vs day one were found in older adults completing a similar protocol (Notley et al. 2018a). These observations are of particular relevance as they indicate that our current findings may reasonably translate to settings in which extended work is performed on multiple successive days (i.e. as is common in many industrial sectors) however, further investigation is warranted to confirm the long-term impacts of heat stress exposure on physical work capacity across a range of working populations. Finally, Kampmann and Bröde (2022) compared indicators of heat strain during the first and third hour of work at a constant workload, performed in a range of climates. They observed significantly lower heat strain (heart rate, rectal temperature) estimates based on the first hour data versus the third hour, and warn that using short studies to develop heat strain prediction models risks underestimating the heat strain for a full work shift, even at moderate heat loads. Whilst such an underestimation of the impact of a longer (up to 6 h over a 7-h shift) work period compared with a shorter 1-h experimental exposure is also evidenced in the present experimental data (Fig. 4, Table S2), it does seem that the impacts of this shift length on physical work capacity for WBGT values up to 30 °C are smaller in the present data than for the impacts on physiological heat strain indicators in the Kampmann and Bröde (2022) data. Whilst in the present study the core temparature in the third hour is between 0.1 and 0.4 °C (cool to most extreme condition) higher than in the first hour, the difference is less than 0.1 °C for all conditions in the the fourth hour immediately after lunch, and then increases again to 0.2–0.5 °C in the sixth hour. The main reason for this difference in magnitude between these studies is the different choice of work paradigm. Kampmann and Bröde (2022) use a fixed workload, which implies that cardiovasular strain—almost by definition—will increase over time, where the present study uses a pacing strategy that limits cardiovascular strain by lowering the workload over time (i.e. Kampmann and Bröde’s data represent work where the pace is fixed, whilst the present study emulates workers self-pacing to limit the total strain). It is clear however, that in both studies, physiological strain levels do increase significantly with longer work periods, and when deducing strain limits, short studies risk underestimating physiological strain. On the other hand it should be noted that in both studies very few absolute core temperatures above 38.5 °C were observed so, on an individual basis, risk remained low.Our findings indicate that when ad libitum water consumption is restricted to rest breaks between work cycles, a progressive water deficit increases by ~ 0.74 L for every 1 L of sweat secreted once WBSL exceeds ~ 2 L. A very similar disassociation of sweat loss and fluid intake was observed previously in German miners exposed to comparable environmental heat stress during an 8-h shift (Kalkowsky and Kampmann 2006), and is consistent with previous reports of the high prevalence of hypohydration in occupations prone to heat stress (Piil et al. 2018). The water deficit that developed across the extended work shift likely impacted PWC via two pathways: (1) a hypohydration-induced reduction of plasma volume (hyperosmotic hypovolemia) leading to a restriction of blood supply available for delivery to active musculature and (2) a blunting of sudomotor responses (i.e. sweating), resulting in reduced evaporative heat loss and, thus, additional heat storage. However, it is acknowledged that that these outcome variables were not directly assessed in the current study, and thus, these potential explanations remain speculative. Nonetheless, these findings reiterate the importance of prescribing appropriate fluid replacement strategies during extended work to avoid the insidious development of hypohydration and help protect worker safety and preserve productivity.

The correction equations presented in this paper (Table 2) are intended to refine our previous estimate of PWC using 1-h work simulations (Foster et al. 2021b). Correction equations are available in which models can be corrected based on WBGT, UTCI, Wet-bulb Temperature, Heat Index, or Humidex. Whilst estimates were good in all models, it should be noted that the UTCI can also predict PWC with added solar radiation (Foster et al. 2021c) and can account for the dynamic effect of wind speed (Foster et al. 2021a), where other indices struggle to capture these two impacts correctly. Taken together, it appears that the UTCI provides the most accurate representation of heat stress compared with all additional heat stress indices used in this study. This finding is not surprising given the UTCI is based on the most complex thermo-physiological model available, at least at the time of writing this paper (Fiala et al. 2012).

## Limitations

A potential limitation of the current study was that rest breaks between work cycles were taken in temperate conditions (21 °C, 50% RH), which is common in many, but not all hot workplaces (e.g. outdoor workplaces). It is acknowledged that this may limit the translational relevance for occupational settings in which such respite from the heat is not always possible. However, in many industries, workers have access to cooler spaces in which rest breaks may be taken, for example, airconditioned staff rooms, areas with additional shade or at bespoke cooling stations, etc. It is, however, expected that the thermo-physiological impact—and thus the observed PWC decrements —would have been more severe if participants had been subjected to heat stress throughout the entire experimental protocol. Secondly, it should be noted that there may be a disconnect between heat-safety recommendations designed to protect against potentially dangerous rises in body core temperature, and those relating to the likely impact of extreme heat on worker productivity/performance. However, during the current fixed heart rate protocol—which in turn limited metabolic heat production—participants rarely approached a body core temperature that might be considered ‘dangerous’, and thus, the concepts of worker safety and worker productivity may not be mutually exclusive. Thirdly, whilst participants were observed across an extended period (~ 7 h), it remains unknown if similar outcomes would have presented across multiple consecutive days of repeated heat exposure. Notley and colleagues (2018) reported an impairment of heat loss in older adults, but not younger adults on the day after prolonged work in the heat. Therefore, it is unclear if a similar phenomenon might have been observed if our participants had performed extended work on consecutive days, potentially resulting in an additional degradation of PWC. Future research is warranted to further elucidate the effects of extended heat exposure across multiple consecutive days on human thermoregulation and work performance. An additional limitation was that only young, healthy males were recruited for study participation. Whilst it is acknowledged that greater heterogeneity exists in the global workforce, it is now well-established that both females and older individuals of working age respond to heat stress in a manner comparable to their younger male counterparts, especially when individual factors (e.g. fitness levels and anthropometrics) are considered (Cramer and Jay 2015; Havenith et al. 1995; Havenith and van Middendorp 1990). The current cohort of male participants exhibited a wide range of fitness levels (VO_2peak_ = 40.7 to 64.0 (mL·kg^−1^·min^−1^)), and we are, therefore, confident that our data are translatable to wider working populations; however, future research is encouraged to confirm this assertion.

Finally, it should be noted that the choice of work intensity at 130 beats·min^−1^ implied that participants were working close to the maximum level sustainable. Whilst perceived effort did not show excessive rises and remained between ‘light’ and ‘somewhat hard’, it is to be confirmed whether this could be sustained for multiple days. Anecdotally, participants were heavily fatigued upon completion of the trial, such that this work level is unlikely to be tolerated on a day-to-day basis. Future research on multi-day work would need to consider this.

## Conclusion

Physical work capacity reduces even in a cool climate when the work is extended from 1 to 6 1-h cycles, with the biggest drop occurring after the lunch break and meal consumption. Relative to the work output in the cool climate, PWC in the heat was further reduced as a function of both the heat stress severity and exposure duration, with a progressive reduction of PWC observed with repeated cycles of work. The magnitude of this cumulative degradation of PWC was augmented by increasing heat stress levels and was linked to a concomitant increase in thermo-physiological strain. Based on these findings, correction equations were developed for use with previously published PWC prediction equations based on a single 1-h work cycle (Foster et al. 2021a, b, c, d), translating these to longer exposures with up to six work cycles, and given the linear expression of the effect, potentially up to 8 cycles. The current findings extend our understanding of the consequences of extended occupational heat exposure and provide empirical evidence and quantification with which the socio-economic burden of future extreme heat may be predicted.

## Supplementary Information

Below is the link to the electronic supplementary material.

Supplementary file1 (DOCX 292 KB)
