# Supplementary material for: Quantifying the impact of heat on human physical work capacity; part IV: interactions between work duration and heat stress severity
Source: Int J Biometeorol. 2022 Oct 5;66(12):2463–76. doi: 10.1007/s00484-022-02370-7 (PMC9684271; doi:10.1007/s00484-022-02370-7)
Supplement: Supplementary file 1 — Supplementary file1 (DOCX 292 KB) [file 484_2022_2370_MOESM1_ESM.docx]

**Supplementary material**

**Figure S1.** Individual (lines) and average (boxes) rectal and skin temperature responses (averaged over the work cycle) to work cycle 1 and 6 in different WBGT conditions. Data presented are the average of the work bout. ns = not significant (paired t-test), * = *p* < 0.05, ** = *p* < 0.01, *** = *p* < 0.001.

**Table S1.** Absolute net energy expenditure across repeated work cycles, cumulative absolute net energy expenditure, relative net energy expenditure compared with net energy expended during work cycle 1, and net energy expenditure expressed relative to the energy expended in the cool reference climate. All data (mean ± SD) presented are for repeated work cycles across the day performed in different environmental conditions.

| **WBGT** | **W1** | **W2** | **W3** | **W4** | **W5** | **W6** |  |
| --- | --- | --- | --- | --- | --- | --- | --- |
|  | ***Absolute Net Energy Expenditure (kJ)*** | | | | | |  |
| **12.6°C** | 1724 ± 378 | 1750 ± 386 | 1629 ± 404 | 1341 ± 404 | 1466 ± 477 | 1483 ± 396 |  |
| **29.4°C** | 1416 ± 336 | 1365 ± 314 | 1206 ± 332 | 1054 ± 320 | 1116 ± 320 | 1071 ± 334 |  |
| **33.4°C** | 1135 ± 331 | 1050 ± 245 | 926 ± 251 | 798 ± 300 | 814 ± 310 | 726 ± 290 |  |
| **36.1°C** | 670 ± 261 | 410 ± 138 | 343 ± 126 | 287 ± 138 | 221 ± 111 | 216 ± 134 |  |
|  | ***Cumulative Absolute Net Energy Expenditure (kJ)*** | | | | | |  |
| **12.6°C** | 1724 ± 378 | 3473 ± 759 | 5103 ± 1160 | 6443 ± 1601 | 7909 ± 2046 | 9392 ± 2438 |  |
| **29.4°C** | 1416 ± 336 | 2781 ± 644 | 3986 ± 964 | 5041 ± 1272 | 6156 ± 1623 | 7227 ± 1964 |  |
| **33.4°C** | 1135 ± 331 | 2185 ± 564 | 3112 ± 784 | 3909 ± 1074 | 4723 ± 1366 | 5449 ± 1642 |  |
| **36.1°C** | 670 ± 279 | 1029 ± 421 | 1373 ± 481 | 1659 ± 594 | 1880 ± 700 | 2097 ± 828 |  |
|  | ***Relative Net Energy Expenditure Compared with Cycle 1 (%)*** | | | | | |  |
| **12.6°C** |  | 102 ± 5 | 94 ± 6 | 76 ± 11 | 84 ± 10 | 85 ± 7 |  |
| **29.4°C** |  | 80 ± 7 | 69 ± 8 | 60 ± 7 | 63 ± 8 | 61 ± 7 |  |
| **33.4°C** |  | 95 ± 13 | 84 ± 17 | 69 ± 12 | 72 ± 16 | 64 ± 15 |  |
| **36.1°C** |  | 65 ± 19 | 55 ± 18 | 43 ± 10 | 34 ± 10 | 32 ± 8 |  |
|  | ***Net Energy Expenditure Relative to the Cool Climate (%)*** | | | | | |  |
| **29.4°C** | 82 ± 7 | 80 ± 7 | 78 ± 6 | 78 ± 6 | 78 ± 6 | 77 ± 6 |  |
| **33.4°C** | 65 ± 10 | 63 ± 8 | 61 ± 9 | 61 ± 10 | 60 ± 10 | 58 ± 10 |  |
| **36.1°C** | 39 ± 10 | 30 ± 8 | 28 ± 5 | 27 ± 5 | 25 ± 5 | 23 ± 5 |  |

| **Table S2.** Average core and skin temperature responses across repeated work cycles in different environmental conditions. All data are presented as mean ± SD. | | | | | | | |
| --- | --- | --- | --- | --- | --- | --- | --- |
|  |  |  |  |  |  |  |  |
| **WBGT** | **W1** | **W2** | **W3** | **Lunch** | **W4** | **W5** | **W6** |
|  | ***Average Core Temperature (°C)*** | | | | | | |
| **12.6°C** | 37.58 ± 0.24 | 37.77 ± 0.22 | 37.70 ± 0.20 | 37.02 ± 0.28 | 37.49 ± 0.15 | 37.84 ± 0.16 | 37.73 ± 0.14 |
| **29.4°C** | 37.36 ± 0.16 | 37.58 ± 0.18 | 37.62 ± 0.16 | 37.18 ± 0.21 | 37.42 ± 0.11 | 37.72 ± 0.18 | 37.71 ± 0.10 |
| **33.4°C** | 37.47 ± 0.21 | 37.83 ± 0.15 | 37.85 ± 0.25 | 37.33 ± 0.22 | 37.52 ± 0.09 | 37.91 ± 0.24 | 37.99 ± 0.22 |
| **36.1°C** | 37.62 ± 0.23 | 38.04 ± 0.24 | 38.00 ± 0.37 | 37.47 ± 0.21 | 37.69 ± 0.20 | 38.03 ± 0.29 | 37.96 ± 0.41 |
|  | ***Average Skin Temperature (°C)*** | | | | | | |
| **12.6°C** | 27.83 ± 1.30 | 27.06 ± 1.49 | 26.89 ± 1.67 | 30.33 ± 2.59 | 27.80 ± 1.39 | 27.28 ± 1.30 | 27.20 ± 1.42 |
| **29.4°C** | 34.42 ± 0.38 | 34.33 ± 0.77 | 34.59 ± 0.93 | 32.43 ± 0.76 | 34.79 ± 0.51 | 34.67 ± 0.76 | 35.01 ± 0.78 |
| **33.4°C** | 35.86 ± 0.20 | 35.73 ± 0.68 | 35.94 ± 0.57 | 32.41 ± 2.04 | 36.22 ± 0.48 | 36.11 ± 0.67 | 36.37 ± 0.54 |
| **36.1°C** | 36.75 ± 0.36 | 36.87 ± 0.45 | 36.90 ± 0.59 | 32.31 ± 1.01 | 36.96 ± 0.34 | 36.96 ± 0.41 | 36.25 ± 0.97 |

| **Table S3.** Average sweat loss and water intake across repeated work cycles in different environmental conditions. All data are presented as mean ± SD. | | | | | | |
| --- | --- | --- | --- | --- | --- | --- |
|  | | | | | | |
| **WBGT** | **W1** | **W2** | **W3** | **W4** | **W5** | **W6** |
|  | ***Fluid/Sweat Loss (ml/kg)*** | | | | | |
| **12.6°C** | 5.30 ± 1.87 | 5.79 ± 1.22 | 5.27 ± 1.25 | 3.50 ± 1.49 | 4.84 ± 1.21 | 5.00 ± 1.54 |
| **29.4°C** | 6.89 ± 2.29 | 7.40 ± 2.51 | 7.99 ± 1.96 | 6.46 ± 1.87 | 7.67 ± 2.12 | 6.93 ± 1.37 |
| **33.4°C** | 8.35 ± 1.65 | 10.50 ± 1.76 | 10.36 ± 2.27 | 7.80 ± 1.75 | 9.58 ± 2.34 | 8.43 ± 3.30 |
| **36.1°C** | 9.02 ± 4.33 | 8.83 ± 2.64 | 8.46 ± 4.67 | 7.44 ± 4.96 | 7.55 ± 3.56 | 4.11 ± 2.56^†^ |
|  | ***Fluid/Water Intake (ml/kg)*** | | | | | |
| **12.6°C** | 4.33 ± 2.30 | 4.27 ± 2.22 | 9.12 ± 3.35 | 3.60 ± 1.85 | 4.78 ± 1.29 |  |
| **29.4°C** | 6.39 ± 2.39 | 5.67 ± 2.38 | 12.18 ± 5.24 | 5.89 ± 2.26 | 5.70 ± 1.29 |  |
| **33.4°C** | 5.85 ± 2.58 | 6.55 ± 2.51 | 11.35 ± 5.19 | 5.12 ± 2.23 | 5.56 ± 1.37 |  |
| **36.1°C** | 7.76 ± 2.34 | 8.17 ± 2.43 | 9.67 ± 5.48 | 5.55 ± 2.10 | 4.94 ± 1.59 |  |

† N.B. The sharp decline in sweat loss observed during W6 in the 36.1°C trial is due to participants prematurely terminating work during the final work cycle of the day and thus being removed from the climate chamber.

**Figure S2.** Individual perceptual responses (with summary boxplots) to each heat stress condition and each work cycle.
